# Viral etiologies of lower respiratory tract infections among Egyptian children under five years of age

**DOI:** 10.1186/1471-2334-12-350

**Published:** 2012-12-13

**Authors:** Caroline F Shafik, Emad W Mohareb, Aymen S Yassin, Madgy A Amin, Amani El Kholy, Hanaa El-Karaksy, Fouad G Youssef

**Affiliations:** 1 US Naval Medical Research Unit No.3, Cairo 11517, Egypt; 2 Faculty of Pharmacy, Department of Microbiology and Immunology, Cairo University, Cairo, Egypt; 3 Faculty of Medicine, Department of Clinical Pathology, Cairo University, Giza, Egypt; 4 Faculty of Medicine, Department of Pediatrics, Cairo University, Giza, Egypt

**Keywords:** Egypt, Direct fluorescence assay, Lower respiratory tract infections, Pediatric, Polymerase chain reaction, Respiratory viruses, Shell vial culture

## Abstract

**Background:**

Lower respiratory tract infections (LRTI) are responsible for a considerable number of deaths among children, particularly in developing countries. In Egypt and the Middle East region, there is a lack of data regarding the viral causes of LRTI. In this study, we aimed to identify the relative prevalence of various respiratory viruses that contribute to LRTIs in young children. Although, nucleic acid-based methods have gained importance as a sensitive tool to determine the viral infections, their use is limited because of their prohibitive cost in low-income countries. Therefore, we applied three different laboratory methods, and presented the different virus prevalence patterns detected by each method.

**Methods:**

We collected nasopharyngeal aspirate samples, demographic data and, clinical data from 450 children under five years of age who presented with LRTI at Abou El Reesh hospital in Cairo during a one-year period. To identify the viral causes of the LRTI we used direct fluorescence assay, real-time reverse-transcriptase polymerase chain reaction (rt-RT-PCR), and shell vial culture. We tested for eight major respiratory viruses.

**Results:**

Two hundred sixty-nine patients (59.9%) had a viral infection, among which 10.8% had a co-infection with two or more viruses. By all three methods, respiratory syncytial virus (RSV) was the most predominant, and parainfluenza virus type 2 (HPIV-2), influenza B virus (FLUBV) were the least predominant. Other viral prevalence patterns differed according to the detection method used. The distribution of various viruses among different age groups and seasonal distribution of the viruses were also determined.

**Conclusions:**

RSV and human adenovirus were the most common respiratory viruses detected by rt-RT-PCR. Co-infections were found to be frequent among children and the vast majority of co-infections were detected by nucleic acid-based detection assays.

## Background

Acute respiratory infections (ARI) can be very severe in young children, and ARI account for one-fifth of all deaths in children less than five years of age. Of those mortalities, 70% occur in Africa and Southeast Asia [1]. Approximately one-third of children will develop lower respiratory tract infections (LRTI) within the first year of life [2]. Premature infants and/or those with compromised immune systems are particularly susceptible to developing respiratory syncytial virus (RSV) related LRTI [3].

Pediatric patients with either upper or lower respiratory tract infections are typically treated symptomatically as outpatients. Diagnostic specimens are generally only obtained and tested in hospital settings, and even then treatment is usually initiated without etiologic determination. In the US, about 1-2% of infants presenting with LRTI require hospitalization [4].

In studies conducted in Europe, a pathogen was identified in 60-85% of LRTI cases [5-7]. Viral etiology accounted for 39-62% of the cases while *S. pneumoniae* accounted for 37% of the cases. The viruses identified included RSV in 24-29% of the cases, rhinoviruses in 24%, human parainfluenza viruses (HPIV) in 10%, and human adenoviruses (HAdV) in 7%, influenzaviruses A and B (FLUAV and FLUBV) in 4-5% of the cases [5-8].

The etiology of viral pneumonia in Egypt was described in a study of patients under five years of age published in 1967–68. HAdV, RSV, human parainfluenza viruses 1 through 3 (HPIV-1, HPIV-2, and HPIV-3), and FLUAV infections were diagnosed by seroconversion [9]. In an effort to improve the treatment of patients with respiratory illness, Egypt embarked on formalized physician training in case management of ARI in the 1980s and 1990s [10,11]. Recently, the burden of atypical pathogens as *Chlamydia* and *Mycoplasma* spp. causing LRTI in children was studied in Egypt [12]. HAdV was also detected using serological methods in 30% of the patients [13]. The clinical presentation of RSV and non-RSV infected children were recently compared in Egypt [14]. Moreover, the burden of a number of respiratory viruses in Middle East countries has been described in several studies [15-21]. Nevertheless, there is a dearth of literature and information regarding the viral etiology of respiratory tract infections in pediatric patients in Egypt and Middle East countries.

Respiratory viral diagnostics rely principally on four techniques: virus isolation in cell cultures, antibody detection (serology), antigen detection, and nucleic acid-based molecular methods [22,23]. For rapid results in a clinical setting, virus isolation is not effective because results can take up to 14 days, which is not timely for treatment decisions. Antigen detection assays such as the direct fluorescence assays (DFA) are more commonly used in clinical settings, because same-day results can be obtained. Nucleic acid-based detection methods such as polymerase chain reaction (PCR) are the gold standard in research laboratories because they are very sensitive; however, these are cost prohibitive in many clinical settings, particularly in newly industrialized and developing nations.

In this study, we sought to determine the predominant viral etiologies of LRTI in pediatric patients and to examine the performance of different laboratory diagnostic methods for the detection of these viruses.

## Methods

### Study Design

A prospective study enrolled pediatric patients under five years of age presenting to the emergency room or the outpatient clinic at Abou El Reesh Hospital in Cairo over a one-year period. Children presenting with any combination of cough, difficulty breathing, fever, chest indrawing, and rapid breathing (> 50 respirations/minute for children under one year of age and > 40 respirations/minute for children from one to five years of age) were enrolled. Chest X-ray results (if available) were obtained at the time of admission. The episode was designated as a radiologically confirmed pneumonia case if an area of consolidation and/or pleural effusion was determined on the chest X-ray. Demographic data and clinical symptoms of the enrolled patients were recorded. Signed informed consent was obtained from the parent or guardian. Patients were excluded if they were over five years of age, unable or unwilling to participate, or if they were already enrolled in the study for the same episode of illness.

### Sample collection

Nasopharyngeal aspirates (NPA) from participants were obtained using a mucus trap (ARGYLE™ DeLee, Kendall, MA, USA). The collected volume ranged from 0.5 to 2 ml. Viral transport media (Hank’s Balanced Salt Solution (Gibco, Invitrogen, NY, USA) with 2.5% w/v Bovine Serum Albumin (Sigma, MO, USA), 2% Penicillin/Streptomycin (Gibco, Invitrogen, NY, USA), and 2.5% HEPES Buffer (Gibco, Invitrogen, NY, USA)) was added to each aspirate. The NPAs were immediately placed at 4°C and transferred to the U.S. Naval Medical Research Unit #3 (NAMRU-3) Cairo, Egypt within 48 hours for viral testing. Upon receipt, the samples were divided into two aliquots. One aliquot was used for direct viral testing by the DFA and the second was kept at −70°C for nucleic acid extraction and virus isolation.

### Viral Testing

RSV, HAdV, HPIV-1, HPIV-2, HPIV-3, FLUAV and FLUBV were tested using DFA, real-time reverse-transcriptase polymerase chain reaction (rt-RT-PCR), and shell vial culture (SVC) procedures. Human metapneumovirus (hMPV) was assessed with rt-RT-PCR only.

### DFA

Aliquots were centrifuged at 700xg for 10 minutes at 4°C. The cell pellets containing mucus were mixed vigorously for 30 seconds and washed with 1 ml phosphate buffered saline (PBS) 3–5 times to remove mucus. At the final wash the supernatant was discarded. The remaining cell pellet was resuspended in 150-250 μl PBS and the sample was examined to determine if the minimum concentration of cells were present (100 cells at 20x magnification). The cell suspension was used to prepare an eight-well slide and the wells were stained using Respiratory Panel 1 Direct Immunofluorescence Assay kit (LIGHT DIAGNOSTICS^TM^ Millipore, CA, USA) according to manufacturer’s instructions.

### rt -RT-PCR

The automated MagMAX Express 96 (Applied Biosystems, CA, USA) was used to extract 200 μl of the second stored aliquot according to the Total Nucleic Acid Isolation Kit protocol from Ambion (Ambion, Inc. NY, USA). Each sample was eluted in 80 μl. Rt-RT-PCR was performed according to the Centres for Disease Control and Prevention (CDC) protocols and using reagents provided by the CDC. A sample was considered positive when the cycle threshold was below 36.

### SVC

R-Mix ReadyCells Vials with coverslip (Diagnostic Hybrids Inc. OH, USA) were used for rapid virus isolation according to the manufacturer’s instructions.

### Statistical analysis

To determine the prevalence of viral etiologies at different age strata, patients were divided into four age groups, and the prevalence of each virus was determined for each group. Fisher's exact tests with stepdown Bonferroni correction for multiple comparisons were performed with SAS Version 9.2 (SAS Institute Inc., NC, USA); p < 0.05 was considered significant.

## Results

### Patients' characteristics

To assess the viral etiologies of LRTI in young children, we enrolled a total of 450 patients according to the previously mentioned case definition during the period from November 2006 to December 2007. Clinical and demographic data were available for 448 (99%) of the patients. Almost all cases (90%) were residents of the greater Cairo area. The mean age of children was 1.1 years and the median age was 8 months. Forty percent of the cases were aged six months or younger. Male children constituted 57.4%. Of the 450 patients, 117 (26%) needed supplementary oxygen, 64 (14.2%) were hospitalized, 4 (0.9%) were admitted to the intensive care unit, among whom 2 (0.4%) died (Table 1).

**Table 1:** Demographic and clinical characteristics of pediatric patients with LRTI during 2007

**Patients' characteristics**	**n (%)**
**Age in months (n = 448)**^**1**^	
0-6	184 (41)
7-12	92 (20)
13-24	102 (23)
25-60	70 (15.5)
**Sex**	
Male	259 (57.4)
Female	186 (41.2)
**Geographical area**	
Greater Cairo	406 (90)
Lower Egypt	12 (2.7)
Upper Egypt	22 (4.9)
**Clinical symptoms (n = 447)**^**1**^	
Cough	438 (98)
Difficulty in breathing	415 (93)
Chest indrawing	339 (76)
Fever	325 (73)

^1^ the number of cases who provided answers to the questionnaire.

### Prevalence of respiratory viruses among patients

**Figure 1:**
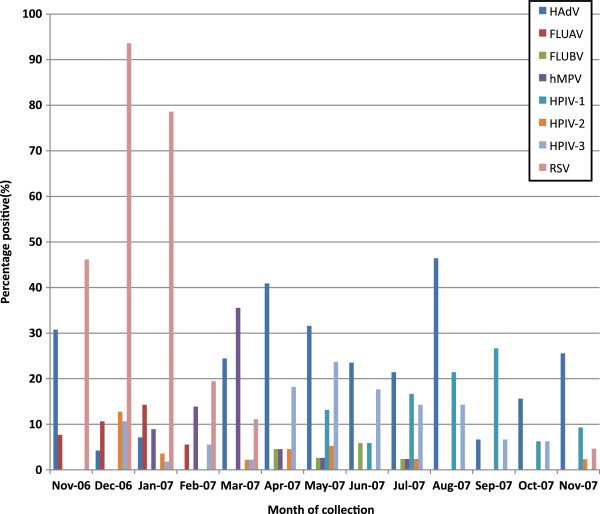
**Monthly distribution of respiratory viruses causing LRTI in children during the study period.** The percentages were calculated by dividing the number of samples positive for each virus by the number of samples collected during each month.

At least one respiratory virus was detected in 269 (59.9%) of cases, and a total of 324 viruses were detected. Co-infection with multiple viruses occurred in 10.8% of the participants. Table 2 shows the viral etiology of the enrolled patients. All but three co-infections were detected by rt-RT-PCR. The seasonal distribution of the viruses during the study year is shown in Figure 1. HAdV could be detected throughout the year, and peaked in April and August, while RSV could only be detected from November through mid February.

**Table 2:** Viral etiology of patients

**Viral etiology**	**Number of patients**
**Single viral agent**	
HAdV	59
FLUAV	7
FLUBV	3
hMPV	20
HPIV-1	23
HPIV-2	1
HPIV-3	22
RSV	85
**Dual infection**	
HAdV + hMPV	6
HAdV + HPIV-1	1
HAdV + HPIV-2	2
HAdV + HPIV-3	6
HAdV + FLUAV	1
HAdV + RSV	3
FLUAV + HPIV-2	1
FLUAV + HPIV-3	1
FLUAV + RSV	6
FLUBV + HPIV-3	1
hMPV + HPIV-2	1
hMPV + RSV	1
HPIV-1 + HPIV-2	2
HPIV-1 + HPIV-3	3
HPIV-2 + RSV	5
HPIV-3 + RSV	3
**More than 2 viral agents**	
HAdV + FLUBV + HPIV-3	1
HAdV + hMPV + RSV	1
HAdV + HPIV-1 + HPIV-3	1
HAdV + HPIV-3 + RSV	1
HPIV-2 + HPIV-3 + RSV	1
HAdV + HPIV-2 + HPIV-3 + RSV	1
NEGATIVES	172
TOTAL PATIENTS	450

**Figure 2:**
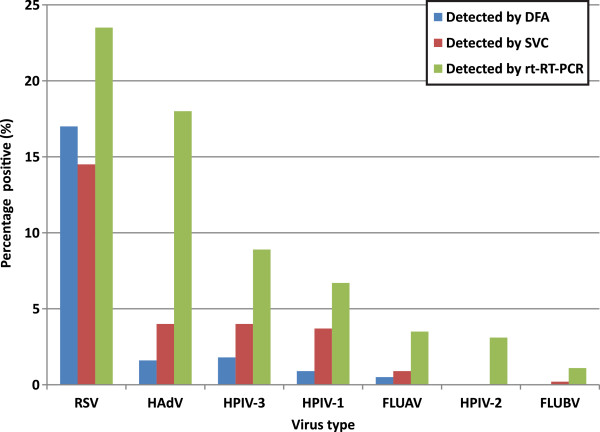
**The percentage positive of each virus detected by each laboratory method showing the relative prevalence of each virus if a particular method was used.** The percentages were calculated by dividing the number of samples positive for each virus by the total number of samples tested by each of the three methods.

In this study, we used three different techniques to diagnose viral infections. The trend of the most predominant and the least predominant viral causes were maintained. However, the relative percentage of cases detected with each method varied. Rt-RT-PCR was the most sensitive for almost all tested viruses. Using rt-RT-PCR as the gold standard, depending on the virus examined the sensitivity of DFA varied from 0% to 77.8% and the specificity varied from 99% to 100% (Additional file 1). Using rt-RT-PCR as the gold standard, depending on the virus examined the sensitivity of SVC varied from 0% to 60% and the specificity varied from 99 to 100% (Additional file 2). The DFA results implicated that HAdV and the HPIV-3 were equally prevalent among patients, and were the second most prevalent viruses following the RSV. On the other hand, the SVC results showed that HAdV, HPIV-3 and HPIV-1 were equally prevalent and followed the RSV in prevalence (Figure 2).

The combined results of the three methods showed that RSV was found in 23.8% of the cases (Table 3), with 34.8% of children under six months old positive for RSV. Compared to other tested viruses RSV was significantly more common in this age group (p < 0.0001). HAdV had an overall prevalence of 18.4% among patients. HAdV was the most frequently detected virus among children aged from 7–12 months, significantly higher than FLUBV, HPIV-1, or HPIV-2 (p < 0.003). Among the same age group, RSV was the second most prevalent virus, and was significantly more prevalent than FLUBV, HPIV-1, or HPIV-2 (p < 0.02). HAdV was also the most frequently detected virus among children aged 13–24 months old, and was significantly higher than FLUAV, FLUBV, HPIV-1, HPIV-2, or hMPV (p < 0.04). Among the oldest age group (25–60 months), HAdV had the highest prevalence, and was significantly higher than FLUAV, FLUBV, or hMPV (p < 0.03). Among this age group, RSV was the second most prevalent virus, and was significantly higher than FLUAV or FLUBV (p < 0.03).

**Table 3:** Distribution of individual respiratory pathogens (n = 324) detected in 450 children

**Age in months**^**1**^	**RSV n(%)**	**HAdV n(%)**	**HPIV-1 n(%)**	**HPIV-2 n(%)**	**HPIV-3 n(%)**	**hMPV n(%)**	**FLUAV n(%)**	**FLUBV n(%)**
**0-6, (n = 184)**	64 (34.8)	18 (9.7)	12 (6.5)	2 (1.08)	11 (5.9)	11 (5.9)	2 (1.08)	0 (0)
**7-12, (n = 92)**	18 (19.5)	21 (22.8)	5 (3.2)	2 (2.17)	10 (10.8)	10 (10.8)	7 (7.6)	1 (1.08)
**13-24, (n = 102)**	12 (11.7)	27 (26.4)	7 (6.8)	3 (2.9)	13 (12.7)	5 (4.9)	7 (6.8)	3 (2.9)
**25-60 , (n = 70)**	13 (18.5)	17 (24.2)	6 (8.5)	7 (10)	6 (8.5)	3 (4.2)	0 (0)	1 (1.4)
**Total positive (n = 450)**^2^	107 (23.8)	83 (18.4)	30 (6.6)	14 (3.1)	40 (8.9)	29 (6.4)	16 (3.5)	5 (1.1)

^1^ Cases were not added up to n due to missing answers.

^2^ Percentages do not add up due to viral co-infections.

Of the 450 patients tested, 75% were sampled within the first week post-onset of symptoms. Half of these were collected in the first three days post-onset. One quarter of the patients were sampled from day 8 to > 4 weeks post-onset of symptoms. Compared to DFA or SVC, rt-RT-PCR demonstrated superior sensitivity for viral detection at all time points after symptom onset (Data not shown).

## Discussion

Determining the etiology of LRTIs in children has long been of interest to the research and clinical community. Viruses have been shown to be the causative agent in 36-85% of LRTIs among children [5-7,21,24]. Different sampling techniques, detection methodologies, and geographical areas can greatly influence the observed burden from each virus. In our study, we tested for eight of the most common respiratory viruses using three popularly used methodologies and identified at least one virus in 59.9% of the cases. Consistent with results of studies conducted in other countries, among the 324 viruses detected, RSV was the most common viral agent among children under five years of age, followed by HAdV; FLUBV was the least common virus detected [2,21,25-27].

DFA has been widely used in the clinical settings because of the high specificity and rapid results. However, DFA is not as sensitive as nucleic acid-based molecular methods. In our study, 22% of samples had a detectable virus by DFA, which is slightly lower than the 32-63% reported by other studies using an indirect immunofluorescence assay [28,29]. Our DFA results indicate that RSV accounted for 17.6% of all the LRTI, followed by HPIV-3 (1.8%) and HAdV (1.6%); these percentages are on the low end of the ranges reported by other studies using similar methodologies [21,28,29]. FLUAV contributed to 0.5% of viruses detected using DFA, while other studies reported rates of 2-15% [28,29]. This difference may represent a true difference in FLUAV burden between the two studies, particularly because the Tang et al. and Zhang et al. studies tested children up to 16 years of age. Other possibilities exist, such as the different sensitivities of the slightly different methods used or year-to-year variations in FLUAV prevalence. Our findings for HPIV-1 (0.9%), HPIV-2 (0%) and FLUBV (0%) using DFA were similar to another published study that reported rates of HPIV-1 (0.6%), HPIV-2 (0.1%) and FLUBV (0.2%) [28]. In contrast, other studies have reported higher detection levels of these three viruses, which could be due to differences in study populations, sensitivity of the assays, or sample quality. Finally, although the detection of co-infections using the immunofluorescence assay was reported [28,29], we did not identify any co-infections with DFA, even though we detected co-infections using other methods. The SVC system is a recently developed method using R-Mix™ cells, and decreases viral detection times from 12–14 days for conventional methods to 24–72 hours [30]. Moreover, some studies demonstrated that the R-Mix™ SVC method is more sensitive for respiratory viruses detection than conventional cell culture, and does not significantly increase laboratory virus isolation costs [31,32]. Our study identified a virus in 26.7% of the patients using the R-Mix™ SVC, which is comparable to a Malaysian study reporting 22% positive by conventional isolation in MDCK, Vero, and Hep-2 cell lines [33]. Using the SVC method, RSV was the most common virus isolated, followed by HPIV-1 and HPIV-3, which is consistent with results using traditional virus isolation methods [33]. LaSala et al. reported that the R-Mix™ system had a low sensitivity for HAdV detection [30] which our results also confirm (Additional files 2). The higher FLUAV prevalence reported by the Malaysian study could be due to the different age group enrolled in the Malaysian study (0–24 months). In both studies, FLUBV was the least common virus among young children with LRTI. Using the SVC system, RSV was successfully isolated. However, better results might have been obtained if the samples were directly inoculated into vials without freezing and thawing, because this is particularly detrimental to the RSV infectivity.

The rt-RT-PCR method detected a viral agent in 59.3% of the participants, which is similar to other studies (35-66%) that used nucleic acid-based techniques [26,34,35]. RSV is the most predominant virus among LRTI patients using rt-RT-PCR, DFA, and SVC, in agreement with studies worldwide [25,26,36]. Our study demonstrated that HAdV, detected in 18.5%, is the second most common causative viral agent for LRTI. Similar observations were reported by other studies [26].

Although for many years virus isolation was the gold standard method to diagnose respiratory virus infections, molecular methods have demonstrated superior viral detection sensitivity. Virus isolation remains an important aspect of virus detection because it is the only means of obtaining a viable infectious virus for further characterization. Isolation alone greatly underestimates the prevalence of respiratory viruses, based on results from nucleic acid detection methods used in this study. This is particularly true for viruses that do not grow well in culture or are highly susceptible to freeze/thaw cycles. For instance, in this study, the rate of prevalence for HAdV is greatly underestimated by DFA (1.6%) and SVC (3.6%) compared to rt-RT-PCR (18.6%). Studies that used a different laboratory technique for each virus had high detectable rates for viruses identified by PCR compared to viruses identified using DFA or virus isolation [21]. This difference should be considered when designing surveillance studies to estimate the burden of viral etiologies of respiratory diseases.

The greater the number of days between symptom onset and sample collection, the more difficult it is to detect a causative agent. Most respiratory viruses are present in high titers in the respiratory tract in the first three days after symptom onset, whereas viral nucleic acid may remain for longer periods. Therefore, isolation-based methods such as SVC loose sensitivity after the first three days post-onset of symptoms, and DFA is similarly affected when viral titers decrease over the course of infection. In contrast to SVC and DFA, rt-RT-PCR remains a sensitive method for virus detection even after two weeks after symptom onset. The high sensitivity of rt-RT-PCR means it can detect a very low titer virus, or viral nucleic acid long after the virus has disappeared, making it difficult to determine if the detected virus is the primary contributor to disease. Thus, nucleic acid detection results must be interpreted with caution, particularly if the sample was taken late after symptom onset [37].

With the development of the PCR scientists were able to detect co-infections at a level not previously possible [26,34,38]. One caveat of this approach is that it is unclear which virus(es) are contributing to disease. By virtue of the nucleic acid-based assay, there is no competition for detection of the various etiologies (unlike SVC) and the amplification step enables detection at lower quantities (unlike DFA). Consequently, PCR is the most useful method to detect co-infections representing near-past and current infections, because of its ability to detect very low viral titers and/or lingering nucleic acid still present later in the infection course. Several common or newly identified respiratory viruses were not assessed in this study, such as picornaviruses, coronaviruses, bocaviruses and newly discovered polyomaviruses, so their contribution to respiratory disease etiology and rates of co-infection in Egypt remain unknown.

## Conclusions

We identified a viral etiology in 59.9% of cases of LRTI in children aged five years and under in Egypt. RSV and HAdV were the most commonly detected viruses in this study.

## Abbreviations

LRTI: Lower respiratory tract infection; RSV: Respiratory syncytial virus; FLUAV: Influenza virus A; FLUBV: Influenza virus B; HPIV: Human parainfluenza viruses; HAdV: Human adenoviruses; DFA: Direct fluorescence assays; PCR: Polymerase chain reaction; hMPV: Human metapneumovirus; SVC: Shell vial culture; rt-RT-PCR: Real-time reverse-transcriptase polymerase chain reaction; PBS: Phosphate buffered saline.

## Competing interests

The authors declare that they have no competing interest.

## Authors' contributions

CFS: Tested all samples by all three methods, analyzed data and drafted the manuscript. EWM: Provided technical oversight throughout the study. Reviewed and edited the manuscript. ASY: Provided advice and analysis of the work results and participated in drafting the manuscript. MAA: Provided supervision of the work and advice in writing the manuscript. AEK: Responsible for patient enrollment according to case definition and manuscript revision. HEK: Preparation of the research proposal, patient assessment, supervision of patient selection, and manuscript revision. FGY: Initiated the research and provided the study design and training of the clinicians at the start of the study. All authors read and approved the final manuscript.

## Pre-publication history

The pre-publication history for this paper can be accessed here:

http://www.biomedcentral.com/1471-2334/12/350/prepub

## Supplementary Material

Additional file 1 Sensitivity and specificity of DFA vs PCR. Click here for file

Additional file 2 Sensitivity and specificity of SVC vs PCR. Click here for file
