# Supplementary material for: Viral etiologies of lower respiratory tract infections among Egyptian children under five years of age
Source: BMC Infect Dis. 2012 Dec 13;12:350. doi: 10.1186/1471-2334-12-350 (PMC3538156; doi:10.1186/1471-2334-12-350)
Supplement: Additional file 1 — Sensitivity and specificity of DFA vs PCR. [file 1471-2334-12-350-S1.pdf]

Sensitivity and specificity of DFA vs PCR

HAdV DFA vs. PCR (n=434)

	PCR POSITIVE	PCR NEGATIVE
DFA Positive	8	0
DFA Negative	79	347

Sensitivity:	9.1%
Specificity:	100.0%
PPV:	100.0%
NPV:	81.4%

FLUAV DFA vs. PCR (n=435)

	PCR POSITIVE	PCR NEGATIVE
DFA Positive	2	0
DFA Negative	13	420

Sensitivity:	13.3%
Specificity:	100.0%
PPV:	100.0%
NPV:	96.9%

FLUBV DFA vs. PCR (n=435)

	PCR POSITIVE	PCR NEGATIVE
DFA Positive	0	0
DFA Negative	4	431

Sensitivity:	0.0%
Specificity:	100.0%
PPV:	NaN
NPV:	99.0%

HPIV-1 DFA vs. PCR (n=435)

	PCR POSITIVE	PCR NEGATIVE
DFA Positive	4	0
DFA Negative	26	405

Sensitivity:	13.3%
Specificity:	100.0%
PPV:	100.0%
NPV:	93.9%

HPIV-2 DFA vs. PCR (n=435)

	PCR POSITIVE	PCR NEGATIVE
DFA Positive	0	0
DFA Negative	12	423

Sensitivity:	0.0%
Specificity:	100.0%
PPV:	NaN
NPV:	97.0%

HPIV-3 DFA vs. PCR (n=434)

	PCR POSITIVE	PCR NEGATIVE
DFA Positive	7	1
DFA Negative	31	395

Sensitivity:	18.0%
Specificity:	99.0%
PPV:	87.5%
NPV:	92.7%

RSV DFA vs. PCR (n=427)

	PCR POSITIVE	PCR NEGATIVE
DFA Positive	74	1
DFA Negative	21	330

Sensitivity:	77.8%
Specificity:	99.6%
PPV:	98.6%
NPV:	94.0%
