# Supplementary material for: Viral etiologies of lower respiratory tract infections among Egyptian children under five years of age
Source: BMC Infect Dis. 2012 Dec 13;12:350. doi: 10.1186/1471-2334-12-350 (PMC3538156; doi:10.1186/1471-2334-12-350)
Supplement: Additional file 2 — Sensitivity and specificity of SVC vs PCR. [file 1471-2334-12-350-S2.pdf]

Sensitivity and specificity of SVC vs PCR

HAdV SVC vs. PCR (n=427)

	PCR Positive	PCR Negative
SVC Positive	14	2
SVC Negative	73	338

Sensitivity:	16.0%
Specificity:	99.0%
PPV:	87.5%
NPV:	82.2%

FLUAV SVC vs. PCR (n=427)

	PCR Positive	PCR Negative
SVC Positive	4	0
SVC Negative	11	412

Sensitivity:	26.0%
Specificity:	100.0%
PPV:	100.0%
NPV:	97.3%

FLUBV SVC vs. PCR (n=427)

	PCR Positive	PCR Negative
SVC Positive	1	0
SVC Negative	4	422

Sensitivity:	20.0%
Specificity:	100.0%
PPV:	100.0%
NPV:	99.0%

HPIV-1 SVC vs. PCR (n=427)

	PCR Positive	PCR Negative
SVC Positive	17	0
SVC Negative	13	397

Sensitivity:	56.0%
Specificity:	100.0%
PPV:	100.0%
NPV:	96.8%

HPIV-2 SVC vs. PCR (n=427)

	PCR Positive	PCR Negative
SVC Positive	0	0
SVC Negative	14	413

Sensitivity:	0.0%
Specificity:	100.0%
PPV:	NaN
NPV:	96.7%

HPIV-3 SVC vs. PCR (n=427)

	PCR Positive	PCR Negative
SVC Positive	16	1
SVC Negative	23	387

Sensitivity:	41.0%
Specificity:	99.0%
PPV:	94.1%
NPV:	94.3%

RSV SVC vs. PCR (n=427)

	PCR Positive	PCR Negative
SVC Positive	62	0
SVC Negative	40	325

Sensitivity:	60.0%
Specificity:	100.0%
PPV:	100.0%
NPV:	89.0%
